# Micro-Organisms

**Published:** 1883-04

**Authors:** 


### HOMŒOPATHIC PHYSICIAN,

A MONTHLY JOURNAL OF MEDICAL SCIENCE.

If our school ever gives up the strict inductive method of Hahnemann, we are lost, and deserve only to be mentioned as a caricature, in the history of medicine."—CONSTANTINE HERING.

Vol. III. APRIL, 1883. No. 4.

### EDITORIAL.

MICRO-ORGANISMS.—Micro-organisms represent the present fashionable craze in medicine. Medicine has had in every age its pet theories; each in its day was thought to be unmistakably true, and its votaries wondered how any one could be so simple as to believe in any of the exploded doctrines of the past! The theory of to-day is always *the true* one, while that of yesterday is *now* easily seen to be unmistakably silly. No one now doubts that micro-organisms are the concomitants of disease; and the terms bacillus, micrococcus, bacteria, etc., are now household words. Babes at the breast reject their time-honored nourishment until assured that no microbacteria are concealed therein; maidens lament the discovery of the bacillus malariæ, believing it to be the deadly enemy of moonlight wanderings; physicians in society and in sick-room learnedly pronounce these same charmed terms; and when the diagnosis reveals bacteria, the learned physician can conscientiously uncork his germicide. Then, parasiticide in hand, the physician proceeds, like the careful housewife, to destroy the vermin. To what glorious ends does science lead its votaries: vermi-cides, bug-destroyers!!

Homœopathists, who should know better, trying to seem learned, also prate of their germicides, their gargles, and their specifics! They seem to fear their medicines cannot cure diseases which are said to be produced by micro-organisms. "We must do something more than simply prescribe the specific remedy," cry some. "The remedy cannot act while the cause of the disease is present," say others. It is well known that these germs do not exist in a *healthy man.** The sickness of the individual produces the habitat, the proper soil, for their growth; hence they cannot be the cause of the disease. Therefore destroying these germs is really destroying an effect and not the cause of the disease.

Upon diphtheria falls the brunt of this absurd practice. In allopathic hands the practice produces no good results; why, then, should homœopaths adopt it to the neglect of a practice which does offer such great results. In homœopathic hands, allopathic

---

* It is a much disputed question whether any varieties of bacteria may exist in the blood or tissues of a healthy animal. In former years the affirmative was maintained by many, notably Billroth; with improvement of methods and differentiation between organized and unorganized particles the number of such affirmations decreased; and the three most noted observers of the present day—Koch, Pasteur, and Ehrlich—affirm that they have never detected bacteria in a healthy animal. Numerous attempts have been made to decide the question experimentally—by the history of healthy tissues, transferred under precautions against contamination, from the living or dying body to conditions of perfect isolation from bacteria. Such experiments demonstrate that some healthy tissues, at least, contain no organisms capable of inducing putrefaction; as the majority of bacteria are, however, incapable of effecting this process, the failure to putrefy does not necessarily prove the absence of all bacteria. Observation and experiment on the living body would also prove the absence of bacteria from healthy animals. The familiar fact that a dead human fœtus may remain in the mother's body for months or years without putrefaction, as in extra-uterine pregnancy, supports the same conclusion. Indeed, it has been repeatedly demonstrated that certain bacterial species, even when injected in considerable numbers directly into the blood or tissues of the living animal, cannot be found after the lapse of some hours; they appear to suffer the same fate as unorganized particles. Hiller even injected into his own skin some bacteria obtained from putrid flesh, and observed only a slight, transient, local œdema.

This failure of putrefactive and other bacteria to reproduce in healthy tissue seems to indicate their inability to maintain the struggle for existence against the animal cells indigenous to the soil. *For seventy years a man may eat, drink, and breathe the ordinary bacteria, and carry a vast and varied assortment of them in his alimentary canal, without suffering putrefaction;* yet so soon as his component cells are destroyed, generally, as in the death of the animal, or locally, as in the gangrene of a toe, the tissues swarm with these minute organisms.—*Dr. Belfield.*

measures not only bring death to the innocent sufferers, but discredit to the system so disgraced. As a consequence of this adoption of allopathic measures by those who call themselves homœopaths, we see such paragraphs as the following going the rounds of the press:

"The *Official Messenger*, the organ of the Russian Government, issued in St. Petersburg, publishes a decision of the Medical Council of that city to the effect that the homœopathic treatment of diphtheria is misleading and dangerous. It had been given a fair trial in the hospitals."

Every true homœopathist *knows* that the treatment of diphtheria here alluded to *was not homœopathic*. If it had been really homœopathic its success would have overwhelmed the allopaths.

Homœopathic treatment is amply able to cope most successfully with diphtheria and any other disease—yes, even if myriads of micro-organisms are present. But to obtain this success one must practice true Homœopathy and must prescribe for the patient, not for the disease. And a great step is taken toward this end when we learn the relative importance, for prescribing of, the common and the peculiar symptoms of the case before us. The *common* symptoms are those found in almost every case of diphtheria, the *peculiar* symptoms are those which are found only in the case before us, and hence are characteristic of it. Thus, most all cases of diphtheria have a membraneous deposit in the mucous lining of the throat, but in some it is white, in some yellow, in some it glistens, in some it looks dull and dirty. Again, most all cases have pain in the throat, some of one kind, some of another. One patient cannot swallow, another wants to do so all the time. These symptoms are unimportant pathologically; but therapeutically they are of the greatest importance. If one would pay more attention to these minor points and less to the more apparent and common pathological points, one would cure more patients and need have no more silly talk about gargles, germicides, or specifics.

